# Quantum Dot Materials and Optoelectronic Devices

**DOI:** 10.3390/nano15231812

**Published:** 2025-11-29

**Authors:** Yaohong Zhang, Guohua Wu

**Affiliations:** 1 School of Physics, Northwest University, Xi’an 710127, China; 2 Qingdao Innovation and Development Center, Harbin Engineering University, Qingdao 266000, China; 3 College of Materials Science and Chemical Engineering, Harbin Engineering University, Harbin 150001, China

Quantum dots (QDs), representing a paradigmatic class of nanomaterials, have garnered substantial interest in both academic circles and industrial sectors over recent decades. In recognition of their pioneering work in QD discovery and synthesis, Moungi G. Bawendi, Louis E. Brus, and Alexey I. Yekimov received the 2023 Nobel Prize in Chemistry—a testament to QDs’ transformative impact on functional nanotechnology [1]. As zero-dimensional semiconductor structures confined to the nanoscale (comprising hundreds to thousands of atoms), these materials exhibit quantum confinement effects when their physical dimensions approach the excitonic Bohr radius. This quantum size effect enables the precise tunability of electronic transitions through the modulation of particle size, morphology, or chemical composition [2]. Consequently, QDs demonstrate exceptional optoelectronic characteristics including size-dependent bandgap tunability, high photoluminescence quantum yields, broad excitation and narrow emission spectra, and compatibility with solution-based processing techniques [3,4].

Current research on QD materials predominantly concentrates on toxic heavy-metal chalcogenides, such as CdS [5] and PbS [6], as well as metal halide perovskites [7]. Environmental safety considerations have propelled the development of “green” alternatives such as carbon quantum dots (CQDs) [8], CuInS_2_ [9,10], and AgBiS_2_ [11,12]. After decades of intensive engineering, QDs have emerged as versatile platforms with transformative potential across numerous domains, particularly in optoelectronics. For display technologies, their precisely tunable emission wavelengths enable monochromatic primary colors (red/green/blue) of exceptional purity and saturation, facilitating next-generation wide-color-gamut ultrahigh-definition displays [1]. In photovoltaics, QDs enhance light harvesting through broadband solar absorption and multiple exciton generation mechanisms [13] when incorporated into hole transport or interfacial layers of solar cells [14]. Their combination of high responsivity, fast response times, and spectral tunability renders them ideal candidates for photodetectors spanning the near-infrared to shortwave infrared (NIR-SWIR) spectrum [15]. Furthermore, leveraging their expansive surface area alongside tailored optoelectronic attributes, QDs function as efficient photocatalysts for water splitting reactions including hydrogen evolution, pollutant mineralization, and CO_2_ reduction, opening up new avenues for sustainable energy conversion [16].

Although QDs have enabled remarkable progress in optoelectronics, several critical challenges persist for industrial deployment such as limited long-term stability, inherent toxicity from heavy metal components, and prohibitive manufacturing costs. To overcome these hurdles, current research focuses on engineering next-generation QD materials featuring eco-compatible compositions, robust operational durability, and enhanced functional performance. This is being achieved through synergistic innovations in synthetic methodologies, post-treatment processes, and device architecture design—ultimately aiming to harness the full technological potential of QDs across diverse optoelectronic platforms.

This Special Issue comprises eight articles including seven research papers and one review article, focusing on QD or nanoparticle-based materials and their application in optoelectronic devices. The main topics of this Special Issue are as follows: optical absorption on electron quantum-confined states of perovskite quantum dots [17], quantum mechanical analysis based on perturbation theory of CdSe/ZnS quantum-dot light-emission properties [18], optimizing the synthetic conditions of “green” colloidal AgBiS_2_ nanocrystals using a low-cost sulfur source [12], in-depth insights into the effect of hydrophilic–hydrophobic group design in amidinium salts for perovskite precursor solutions on their photovoltaic performance [19], recent advances and strategies in all-inorganic perovskite quantum dot-based blue light-emitting diodes [20], studies on laser-induced multi-exciton generation and dynamics via multi-photon absorption in CdSe quantum dots [21], GaAs cone–shell quantum dots in a lateral electric field: exciton Stark shift, lifetime, and fine-structure splitting [22], and hybrid amino acid ligand-regulated excited dynamics of highly luminescent perovskite quantum dots for bright white light-emitting diodes [23]. This Special Issue will celebrate cutting-edge research on QD materials and optoelectronic devices, engaging the broad readership of *Nanomaterials*.
